# A Case of Extra-Uterine Pregnancy

**Published:** 1840

**Authors:** Samuel Sexton

**Affiliations:** San Augustine, Texas


Art. VIII.—*A case of Extra-Uterine Pregnancy, with an account of the appearances on dissection.* By Dr. Samuel Sexton, of San Augustine, Texas.

On the 9th of June, 1838, I purchased a negro woman said to be twenty-four years old, and warranted sound. Her appearance indicated health until about three months previous to her death on the 19th of August, 1839—when, on examination, a tumor was found in the abdomen, near the umbilicus, as large as the gravid uterus in the seventh month. She continued able to perform her usual labors until within two weeks of her death, except for a few days about her menstrual periods. These, she stated, had been regular since the weaning of her last child, six or seven years before, soon after which she discovered the tumor. Her last child was stillborn—she was married early, and had children in quick succession. She was seized a fortnight before she died, with violent pains in the abdomen, attended with high fever. An examination externally, and per vaginam, did not enable me, or my friend, Dr. Griffith, to determine the character of the tumor. Warm embrocations were applied to it with apparent advantage, aud two days before she died she was walking about apparently comfortable and convalescent. The next day she grew worse, and in twenty-four hours died, the tumor in that time having greatly increased in size.

*Autopsy.*—On opening the abdomen we found a sac large enough to contain a fœtus of six pounds weight, lying anterior to the abdominal viscera. On opening it, we found it to contain a substance answering to the description of the *gras des cadavres*, or *adipocire*, hair, blood, and a piece of bone, probably a portion of the base of the skull, in a perfect state. To the main sac, and communicating with it from the left, was attached a smaller one, which contained the same matter. Other smaller sacs, filled with the same substances, we found, the whole number being five—presenting what appeared to be five extra-uterine fœtations.

The uterus was found in its natural situation, of its usual size, but of unnatural consistence, approaching to cartilage. A part of the round ligaments was in its proper place—traces of the broad ligaments and fallopian tubes were discovered, but forced out of their natural position by the enlargement of one of the ovaria, which seemed to form the larger sac. No traces of the ovaria were found.

The viscera of the abdomen were in a normal state. The five sacs, the uterus and appendages were preserved and may be seen as described.

*September*, 1839.

